# Qualitative assessment of a Context of Consumption Framework to inform regulation of cigarette pack design in the U.S.

**DOI:** 10.18332/tid/82925

**Published:** 2018-02-10

**Authors:** Joseph G. L. Lee, Paige E. Averett, Tiffany Blanchflower, Kyle R. Gregory

**Affiliations:** 1 Department of Health Education and Promotion, College of Health and Human Performance, East Carolina University, Greenville, North Carolina, United States; 2 School of Social Work, College of Health and Human Performance, East Carolina University, Greenville, North Carolina, United States; 3 Department of Interior Design and Merchandising, College of Health and Human Performance, East Carolina University, Greenville, North Carolina, United States

**Keywords:** product packaging, tobacco products, tobacco industry, government regulation, United States Food and Drug Administration

## Abstract

**INTRODUCTION:**

Researchers and regulators need to know how changes to cigarette packages can influence population health. We sought to advance research on the role of cigarette packaging by assessing a theory-informed framework from the fields of design and consumer research. The selected Context of Consumption Framework posits cognitive, affective, and behavioral responses to visual design. To assess the Framework’s potential for guiding research on the visual design of cigarette packaging in the U.S., this study seeks to understand to what extent the Context of Consumption Framework converges with how adult smokers think and talk about cigarette pack designs.

**METHODS:**

Data for this qualitative study came from six telephone-based focus groups conducted in March 2017. Two groups consisted of lesbian, gay, and bisexual participants; two groups of participants with less than four years college education; one group of LGB and straight identity; and one group the general population. All groups were selected for regional, gender, and racial/ethnic diversity. Participants (n=33) represented all nine U.S. Census divisions. We conducted a deductive qualitative analysis.

**RESULTS:**

Cigarette package designs captured the participants’ attention, suggested the characteristics of the product, and reflected (or could be leveraged to convey) multiple dimensions of consumer identity. Particular to the affective responses to design, our participants shared that cigarette packaging conveyed how the pack could be used to particular ends, created an emotional response to the designs, complied with normative expectations of a cigarette, elicited interest when designs change, and prompted fascination when unique design characteristics are used.

**CONCLUSIONS:**

Use of the Context of Consumption Framework for cigarette product packaging design can inform regulatory research on tobacco product packaging. Researchers and regulators should consider multiple cognitive, affective, and behavioral responses to cigarette pack design.

**ABBREVIATIONS:**

FDA: Food and Drug Administration, FSPTCA: Family Smoking Prevention and Tobacco Control Act of 2009

## INTRODUCTION

The passing of the Family Smoking Prevention and Tobacco Control Act of 2009 (FSPTCA) gave the U.S. Food and Drug Administration (FDA) the authority to regulate tobacco products, including premarket review authority for ‘new tobacco products’^1^. Premarket review requires that new tobacco products, not substantially equivalent to a predicated tobacco product, demonstrate that they are appropriate for the protection of public health. This is a robust standard that must take into account users and non-users alike in an attempt to assess the net population-level health impacts of tobacco products^1,2^. Including packaging as part of the premarket review is critical when assessing whether product changes have implications for population health^1,3,4^. Reynolds’s Camel No. 9 brand, for example, with its sleek pink and black packaging, was associated with an upswing in adolescent female smoking^5^. Industry documents show how changes to product packaging have likely resulted in population-level changes in smoking patterns by increasing smoking among women^6,7^. However, FDA attempts to regulate changes to pack design have met with repeated legal challenges by the tobacco industry^8^.

**Figure 1:**
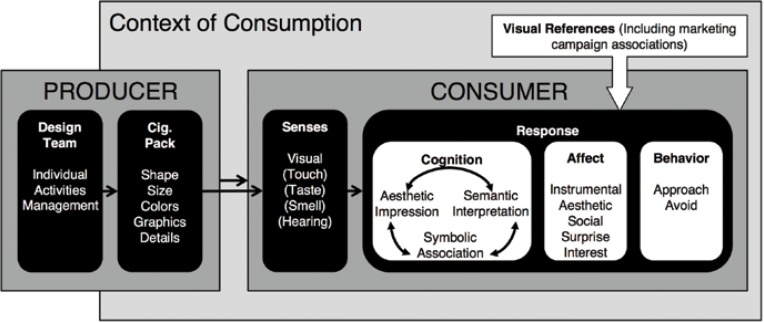
Modified framework for design as a process of communication with visual references Reprinted with modifications from Design Studies, Volume 25, Issue 6, Crilly N, Moultrie J, Clarkson PJ, Seeing things: Consumer response to the visual domain in product design, Page No. 555, Copyright (2004), with permission from Elsevier.

The authority of the FDA hinges on the strength of the scientific literature on how changes to the visual design of cigarette packs can influence consumer responses. It is critical that FDA regulations be able to draw upon scientific evidence designed to inform them^9^. Previous research in the fields of consumer behavior and design has shown that product packaging influences consumer perceptions for a myriad of consumable products^10-12^. Particular to the tobacco-control literature, numerous studies have examined product packaging from specific vantage points, including plain packaging and graphic warning labels^13-16^. However, few studies have focused on pack design among U.S. smokers. Moreover, no research has leveraged a theory-informed framework that could guide researcher understanding of different types of consumer responses to cigarette pack design. The FDA calls for research to inform efforts to ‘[r]estrict product changes to protect public health’ as part of its framework of tobacco regulatory research (Ashley et al.^9^ p. 1047). To answer this call, we draw on an influential Context of Consumption Framework from the field of design by Crilly and colleagues^17^ (Figure 1) that focuses on the visual design of products. Use of such a theory-informed framework could open new areas of research to support science-based regulation of tobacco products.

### Utilizing the Context of Consumption Framework for cigarette pack design research

To introduce the reader to the Context of Consumption Framework, we first use it to organize a brief overview of selected research on the role of cigarette packaging on consumer perceptions. We focus in particular on research based in the U.S. as it provides a unique regulatory context for this work, given the absence of graphic warning labels and preclusion of plain packaging requirements.

As shown in Figure 1, a producer’s design team creates the visual design of a product. Previous research has leveraged tobacco industry documents to describe the internal decisions of the tobacco industry (i.e. the producer) and its own testing of pack characteristics^3,6,18,19^. Our interest, however, is centered on how consumers respond to product design after the product is generated.

The Context of Consumption Framework posits theory-informed consumer responses to the product now created by the design team. These are cognitive, affective, and behavioral responses. While these responses will be recognized as core concepts in psychology^20^, the framework expands these to include a typology of responses that could inform tobacco product regulation.

The Context of Consumption Framework breaks down cognitive responses into three types: **Aesthetic impression** that denotes the appealing nature of the design, as well as its contrast (in its own design and against others) and novelty, linking or contrasting the product to other similar products. In previous research, a cross-sectional study assessed the ability of the pack design to attract attention and rated packs for attractiveness^11^, while an experiment assessed the appeal of women-oriented packaging^21^. Globally, packs have been rated for similar responses, e.g. being ‘worth looking at’^22^. **Semantic interpretation** that denotes the apparent utility of the product and its perceived qualities, assessed in the literature as effectiveness^11^, health^11,12,21^, nicotine^12^, quality^11^, tar^11,12,21^, and taste^11,12,21^. Globally, researchers found evidence to support that packaging communicates information about the cigarettes within (e.g. Ford et al.^22^, Guillaumier et al.^23^). Finally, **Symbolic association** that denotes how the product reflects users’ identities. We identified one study that experimentally tested women-oriented pack designs^21^ and a cross-sectional study that rated packs based on ‘concern with image’^12^. Globally, there are similar findings, including about emerging products^24-26^.

The Context of Consumption Framework suggests another type of response, affective, labeled ‘affect’ in Figure 1. Affective response can be a feeling, mood, or emotion prompted by the product^20^. The Context of Consumption Framework posits five different types of affective responses evoked by the product: **Instrumental**, emotions based on the perception of whether the product will fulfill the consumer’s objectives (e.g. satisfaction); **Aesthetic**, emotions based on how the product might delight or offend (e.g. attraction); **Social**, emotions based on social norms about the product (e.g. admiration); **Surprise**, emotions based on novelty (e.g. amazement); **Interest**, emotions based on the promise and challenge of the product (e.g. fascination). We identified no U.S. studies that have considered affective response to tobacco product pack designs, although affective responses to marketing of alternative tobacco products have been documented^27^.

Evidence about behavior, which describes consumers’ approach or avoidance of a product, largely comes from epidemiological cohort studies and surveys that did not directly assess responses to cigarette packs (e.g. Pierce et al.^5^). Globally, researchers have documented the importance of pack design on behavior^16^.

Thus, the Context of Consumption Framework provides three cognitive, five affective, and two behavioral responses that could result from the visual design of cigarette packs. As evidenced in this brief, selected review, a conceptual framework can identify gaps in the evidence base. For example, we find relatively little work on affective responses to the design of cigarette packs. These gaps, when identified in the U.S., represent weaknesses in the scientific evidence available to develop, implement, and defend strong science-based regulations of tobacco products.

This paper thus aimed to assess the potential for using the Context of Consumption Framework in research on consumer responses to tobacco product packaging. Specifically, we attempt to answer the question: ‘Do smokers’ responses to cigarette pack design align or diverge from the Context of Consumption Framework?’. If smokers’ responses to cigarette pack design show alignment with the Context of Consumption Framework, this would provide evidence that tobacco regulatory-science researchers should consider assessing cognitive, affective, and behavioral responses. Alternatively, if smokers’ responses diverge, this would suggest parts of the Context of Consumption Framework could be omitted in tobacco regulatory-science research or that there may be other responses that should be examined.

## METHODS

This paper is a secondary analysis of qualitative data collected for a research project, funded by the National Cancer Institute and the FDA, entitled ‘Cigarette packaging: design, cognition, and consumer choices’ (R03CA212542)^28^. Following best practices in tobacco regulatory science^29^, our research included a lawyer trained in tobacco regulatory science (KRG) in the entire research process. In March 2017, we created six focus groups with adults who were current smokers. To maximize the geographic diversity of these groups, we conducted interviews of the groups by telephone. Telephone-based focus groups are increasingly being used and come with advantages: including logistics, anonymity of participants, and geographical representation^30,31^. Use of video-based groups would have omitted participants without internet access, and use of in-person focus groups would have omitted participants from many regions of the country. Participants were recruited from the National Opinion Research Center (NORC) at the University of Chicago’s AmeriSpeak Panel, which is a probability-based, nationally representative panel. Participants for the focus groups were purposively recruited. Recruitment screening was conducted by NORC with the attempt to maximize diversity by race, ethnicity, region, sexual orientation, and gender. We included a focus on sexual minority (e.g. lesbian, gay, and bisexual) and low-socioeconomic status smokers, as both groups are at much higher risk of tobacco use than the general population^32^. We did this to ensure that groups at high risk of tobacco use would be well represented in our groups. Two focus groups consisted of sexual minority participants, two focus groups consisted of lower socioeconomic status participants (classified as less than 4 years of college education), one focus group consisted of sexual minority and straight participants, and one focus group consisted of the general population. Participants were compensated by NORC for their participation in AmeriSpeak ‘points’. All groups were moderated by an experienced NORC staff person and lasted 60-90 minutes. Two authors (JGLL, PEA) attended each group and confirmed saturation of themes. Audio was recorded after participants verbally consented. Recordings were professionally transcribed using a smooth verbatim protocol.

We developed a semi-structured focus-group guide designed to elicit thoughts about the meaning and design of cigarette packs, with questions designed to assess the cognitive, affective, and behavioral domains of the Context of Consumption Framework. This guide is available online^28^. We did not include visual stimuli in this study; instead, we asked participants to talk about the design of cigarette packs they had encountered. For example, we used probes such as: ‘Tell us what the cigarette pack of your first cigarette looked like’ and ‘Tell me about a time at any point in your life that “your brand” changed its packaging; how did it make you feel?’. This added to the richness of the discussion, as one participant would ask what a pack that was being discussed by another participant looked like. That is, instead of asking for responses to stimuli provided by researchers, we asked about salient memories and participants’ own packs or packs they recalled. Prior research has examined and confirmed the role of senses, such as touch^33^ in relation to cigarette packs; we thus focused on visual responses. We tested the guide in a pilot focus group of seven people and used the results to inform our probes in the six groups.

### Analysis

As we wished to assess an existing framework, we used a deductive qualitative analysis method^34^. In deductive coding, data are sorted as they fit with existing concepts (or codes) of an *a priori* framework, i.e. the different response types of the Context of Consumption Framework. We coded in four levels of analysis: open coding, axial coding, selective coding, and a negative case analysis. First, in open coding, we coded the text using codes derived *a priori* from the Context of Consumption Framework. Second, in axial coding, we further analyzed data within the Context of Consumption Framework codes. Here, we attended to groupings or sub-themes within the concept to see if further refinement of the Context of Consumption Framework was possible. Third, in selective coding, the data were further examined to see if there was a possible reduction to a single category or core concept. Fourth, a negative case analysis examined data that did not fit with the *a priori* Context of Consumption Framework. This analysis process was conducted by one author (PEA, a professor of social work and qualitative methodologist) and discussed, refined, and verified with a second (JGLL, a tobacco control and public health researcher) at each stage. A third author (TB, a consumer behavior researcher) also refined codes in the selective coding stage. The analysis team included past experience smoking cigarettes and a short phase as a social smoker, but no member of the team currently uses tobacco products. We provide quotes selected for their representativeness of each theme.

We used best practices in qualitative research to ensure the reproducibility of our study^35^. These include credibility, dependability, transferability, and confirmability. To address these, we engaged in triangulation of analysis, an audit trail, and research journal and peer debriefing.

### Participants

The six focus groups had 33 participants of whom 29 fully completed the group, one dropped out after completing most of the group (i.e. 1 hour and 10 minutes), one dropped out after 40 minutes, and two dropped after 20 minutes. Dropping out was not reported to be about the content of the study. Of the 33 participants, 36% identified as LGB, 49% as having less than four years college education, 49% identified as White, 24% as Black, 9% as Hispanic, 3% as American Indian, and 15% as multi-racial. Participants represented all 9 U.S. Census divisions. Six participants (18%) did not have internet access at their homes. All reported current smoking based on 100 cigarettes in lifetime and currently smoking some days (6%) or every day (94%); 46% usually smoked menthol. Fifty-eight per cent smoked their first cigarette of the day within 30 minutes of waking up. Age ranged from 22 to 62 years, with a mean of 46 (sd=11.5), and most participants identified themselves as female (64%).

## RESULTS

Consistent with the Context of Consumption Framework, we found support for the *a priori* themes of Cognition (sub-themes: Aesthetic, Semantic, Symbolic), Affective Response (sub-themes: Instrumental, Aesthetic, Social, Surprise, and Interest), and Behavior. Based on the negative case analysis, we do not find cause to modify or reject any parts of the Framework. Cognitive, affective, and behavioral responses to the visual design of cigarette packs are supported by the data in this study. We did not identify any differences by the composition of the focus groups.

### Cognition

Within the Context of Consumption Framework, after the initial sensory experience, cognition is one key aspect of consumer response. According to the Context of Consumption Framework, cognitive responses are judgments made by the consumer based on provided visual information. The cognition category includes three specific areas of response, as described and discussed below.

*Aesthetic.* Aesthetic impression addresses consumers’ overall judgments about a product’s attractiveness or unattractiveness. Typically, consumers contrast products against one another in terms of novelty or similarity to make aesthetic judgments. We found strong support for the role of attractiveness, for example:

*‘…they look cool because they’re just so bright and different than the rest of the cigarettes such as Pall Mall. You don’t notice those on the shelf but American Spirits you would.’* (Group 2, Lower Education),

*‘The black and gold, my eye just directly goes straight to it.’* (Group 3, LGB).

However, it is of note that the participants did not provide clear descriptions of cigarette packages that were judged as unattractive based on product packaging. Rather, some participants shared their view that they never judged a cigarette pack unattractive. In short, consumers cognitively judged cigarette packaging as overwhelmingly aesthetically attractive and appealing:

*‘I have never really seen anything that visually looked repulsive to me.’* (Group 2, Lower Education).

*Semantic.* Semantic interpretation denotes the perception of the product’s utility or function. For cigarette packaging this can relate to perceived quality, tar levels, taste, etc. Many of the participants discussed the products’ characteristics and how these are perceived through the packaging style. Participants’ discussion supported the idea that packs serve a semantic function, signaling to consumers that some packs were healthier, natural, and basic or cheap in quality:

*‘It just makes me think of free-spirited and it’s considered like the healthier of all the cigarettes because of the less ingredients and what not and the packaging reflects all that.’* (Group 2, Lower Education),

*‘I know for me like the Basic packs, despite the name being Basic, it’s very plain and it just makes me think of inferior quality. The same with other, like, cheaper brands, like just the way the packaging is very simple and so it does make me think that it’s a less quality of a product.’* (Group 5, Lower Education).

*Symbolic.* Symbolic association refers to the consumer’s perception of what product design communicates about the consumer’s personal and/or social identity. Participants discussed particular identities and stereotypes of various smokers. Moreover, they clearly connected these identities and stereotypes with the consumption of cigarettes from packs with particular designs. This aspect of the Context of Consumption Framework seems particularly salient for assessing packaging and its impact upon consumers’ connection of particular designs to specific races, genders, subcultures, and psychographic profiles:

‘Well, to be honest with you I think what would characterize the Marlboro Reds are straight, white men.’ (Group 1, LGB),

‘Well take the Capris for example. They’re marketed generally for women, and there’s a nice really thin, slim, long, you know.’ (Group 4, LGB/Mix).

### Affective response

Within the Context of Consumption Framework, another aspect of consumer response is affective response, which captures consumers’ emotions, feelings, and moods associated with visual design. More specifically, the affective response domain includes five sub-themes: Instrumental, Aesthetic, Social, Surprise, and Interest.

*Instrumental.* Instrumental emotions are tied to consumers’ perception of whether the visual product design signals that a product will assist or hinder their consumption objectives. Typically, these emotions are expressed as satisfaction or, among our participants, as relief:

*‘The only thing I think of whenever I see the actual pack is that I’m going to smoke one and relief is coming.’* (Group 3, LGB).

Discussions about instrumental characteristics occurred mostly when participants were asked about the experience of having a pack change its design, and in these instances participants shared their concern that the changes would be disappointing:

*‘But speaking for myself and the peers that I frequently smoke around and they smoke as well, [the change in Newport packaging after acquisition by Reynolds American] was basically more of a discomfort and it made us kind of feel like something was wrong with the cigarettes.’* (Group 3, LGB).

*Aesthetic.* Aesthetic emotions include disgust or attraction, and are connected with the ability for visual product design to delight or offend a consumer’s senses. In turn, aesthetic emotions elicit positive or negative feelings and moods about a visual design, and ultimately the product. Our participants had very strong feelings when looking at certain cigarette packs. These feelings included disgust, fearfulness, happy, and/or attraction, all of which related to package colors and design.

*‘GPC’s blue pack just almost makes my stomach turn.’* (Group 2, Lower Education),

*‘The American Spirit, the yellow pack, always seemed of sun – it made me think of sunny days, beaches, outside activities.’* (Group 3, LGB).

*Social.* Social emotions include indignation or admiration, and are tied to consumers’ beliefs that the design complies with social norms. The participants spoke of certain brands’ iconic packaging style and that a brand’s history and longevity, as demonstrated through packaging, can evoke esteem or regard among consumers:

*‘I think as far as like a menthol cigarette, I think the way that they use their green coloring, the green and then the white but mostly green was like a power statement as far as a menthol cigarette was concerned.’* (Group 2, Lower Education),

*‘I don’t think the Newport packaging has changed all that much since they’ve been around. It’s pretty much still the same. You kind of can’t miss them. They’re like Marlboro; you kind of can’t miss the easy identification of it.’* (Group 1, LGB).

*Surprise.* Emotions of surprise include amazement and novelty about the design of cigarette packs. The surprise emotion, as expressed by our participants, seems to stand in contrast to the social admiration of long standing iconic brands. Social admiration was based on not changing the design, while surprise is based in atypical design. The surprise sub-theme captures participants’ discussions of cigarette packaging that was found to be appealing due to its unique designs. Participants seem to notice packaging that did not feature standard design elements. For instance, designs were often considered unique when they featured bright colors, contrasting colors, and patterns:

*‘It doesn’t look like a typical pack of cigarettes, as far as the package. I mean, it’s packaged in a typical cigarette box, but the decoration of the box is not typical of a regular cigarette.’* (Group 1, LGB),

*‘Yes, one of my supervisors at work smoke them. It’s like a yellow pack with an Indian with all the little colorful stuff in it. That’s what draw my attention to it. I’m like, what are you smoking?’* (Group 4, LGB/Mix).

*Interest.* The emotions within this area capture participants’ boredom or fascination with pack design and the connection of these emotions to their hopes of product changes. The following quote demonstrates the feeling of fascination and a hint of challenge:

*‘The camel with the whole picture that goes along with it. There’s actually a little thing sometimes you can find and it became like a thing where you could actually have games and find things on the back of the Camel packs and there were things like that and just the way it looked. It was the only one of its kind.’* (Group 2, Low Education).

In contrast, the following quote demonstrates boredom in pack design and that packages are only noticed when they change:

*‘I guess I’m oblivious to what’s on them after a while. You just notice if they change.’* (Group 4, LGB/Mix).

### Behavior

After the cognitive and affective responses, the Context of Consumption Framework posits that consumers select one of two paths: approach or avoid the product. When a consumer enters the approach path they will often engage in one or more of the following behaviors: information search, purchase, and/or consumption of the product. If a consumer selects the avoidance path they will likely ignore the product or fail to purchase it. The participants shared experiences supporting the connection between cigarette packaging, both the approach and avoidance paths:

*‘I actually started smoking them for a little while because I thought the little pack was cute.’* (Group 4, LGB/Mix),

*‘I think that is what the packaging is trying to convey to the consumer, is that hey it’s okay, this is a safer cigarette for you to smoke, and that’s why I picked them up to begin with.’* (Group 6, General Population).

### Negative case analysis

While the participants’ discussion of cigarette pack design confirmed the domains of the Context of Consumption Framework, our participants also clearly expressed that design is not the only driver of behavior. There were substantial discussions on the importance of price and couponing, and our participants, who were smokers and tended to be middle age, were keenly interested in the delivery of nicotine when going into withdrawal. For instance, one participant went as far as to say, *‘as long as it’s not [wrapped in] doo-doo. Okay? It doesn’t matter’* (Group 1, LGB). Yet, others indicated that design mattered in their original decision to try a particular cigarette and that the role of design may be different across the life course:

*‘I think some things are geared as influencing younger people. I don’t think people over 40 get influenced by packaging for cigarettes.’* (Group 1, LGB).

## DISCUSSION

### Principal findings

In the six focus groups of smokers from across the U.S., we found that the Context of Consumption Framework aligned with how smokers discussed responses to the visual design of cigarette packs. Participant discussions yielded data that supported the Cognitive response theme of visual design as well as its Aesthetic, Semantic, and Symbolic sub-themes. Cigarette package designs captured the participants’ attention, suggested the characteristics of the product, and reflected (or could be leveraged to convey) multiple dimensions of consumer identity. Particular to the affective responses to design, our participants expressed that cigarette packaging conveyed how the pack could be used to particular ends (Instrumental), created an emotional response to the designs (Aesthetic), complied with normative expectations of a cigarette (Social), elicited interest when designs change (Surprise), and prompted fascination when unique design characteristics are used (Interest). Participants also tied changes to the visual design of packs to behavior.

### Study findings in context

Our findings fit within the broader qualitative literature about cigarette pack design and marketing. Previous ethnographic research has documented the salience of touch in smokers’ relationship with cigarette packs^33^. Other qualitative studies further affirm the role of cigarette branding, including package design^36^, in youth’s social identities (i.e. the cognitive symbolic domain)^37,38^, and the appeal of packaging^25,39,40^. Reseach into tobacco-industry documents shows careful psychographic targeting by the industry^41^. There is clear evidence that pack designs reference broader marketing imagery^42^. In addition, the size of design elements, such as the Marlboro chevron, have been used to communicate health claims (e.g. tar levels)^43^. Interest in alternative tobacco product marketing is tied to affective responses of hope^27^.

This study is compatible with the broad scientific consensus within consumer behavior that the visual design of product packaging matters to consumers and ultimately influences their perceptions and behaviors^17,44,45-47^. For instance, in the Cognition sub-theme of Symbolic response we found that consumers often associated specific pack designs with a particular type of consumer, often a stereotype associated with a lifestyle choice and/or demographic factors. This can be explained by the self/product congruency literature, which supports the notion that consumers often purchase products that reflect or align with their ideal personal or social self^48^.

This research suggests researchers and FDA regulators should consider multiple types of consumer responses (i.e. cognitive, affective, and behavioral) to the visual design of cigarette packages. Our findings have important implications for measurement of outcomes in experimental and observational research on cigarette packaging. Specifically, the Context of Consumption Framework suggests that researchers and regulators should assess the three cognitive and five affective responses posited by the Context of Consumption Framework.

### Limitations

This study has a number of limitations. First, while the use of a telephone for the focus group allowed broad geographic representation and did not require internet access, it limited face-to-face participant-to-participant interaction, and as such the ability to read facial expressions. Additionally, the phone mode limited our ability to eliminate other distractions for participants, and likely made it easier to drop out of the group. Second, although we recruited from the probability-based AmeriSpeak Panel, our findings reflect qualitative themes that emerged in our unique, purposively sampled set of participants, and as such the findings may not be generalizable to all smokers. Not all identities were represented in our study; for example, we had a limited number of American Indian and Hispanic/Latino participants and no participant identified as Asian. Our participants skewed to an older age demographic, which may have influenced our results. Third, this study solely examined cigarette packaging and did not assess applicability to other tobacco products. Fourth, due to the use of a panel service, we were not able to check our findings with the participants. Fifth, the Context of Consumption Framework contains other sources of influence and potential moderators; future research should examine these for use in tobacco regulatory research.

## CONCLUSIONS

Future research should experimentally link the domains of the Context of Consumption Framework to consumer responses and, ultimately, to changes in population health. We recommend additional work on developing standardized measures of these responses. Our research identifies themes that show compatibility with the Context of Consumption Framework, but does not indicate whether some responses are more influential than others in changing purchase or use behaviors. Future research should extend this to youth and on the role of package design in initiating cigarette smoking.

When asking tobacco manufacturers to demonstrate a new product’s substantial equivalence, the FDA may request additional data comparing consumer perceptions of the new tobacco product and the predicate that could affect initiation, cessation, frequency of use, patterns of use, smoking behavior, and perceptions of harm or addictiveness^49^. Given the potential for visual design changes in cigarette packages to influence population health (e.g. Camel No. 9^5,7,50^), building the scientific evidence for pre-market review of cigarette products remains an important area of tobacco research. Tobacco control researchers and federal regulators should leverage the theory-informed Context of Consumption Framework to assess the impact of product packaging on a full range of consumer responses.
